# The Effect of Dietary Phospholipids on the Ultrastructure and Function of Intestinal Epithelial Cells

**DOI:** 10.3390/ijms24021788

**Published:** 2023-01-16

**Authors:** Snezhanna Saydakova, Ksenia Morozova, Olga Snytnikova, Maryana Morozova, Lidiya Boldyreva, Elena Kiseleva, Yuri Tsentalovich, Elena Kozhevnikova

**Affiliations:** 1 Scientific-Research Institute of Neurosciences and Medicine, 630117 Novosibirsk, Russia; 2 The Federal Research Center Institute of Cytology and Genetics SB RAS, 630090 Novosibirsk, Russia; 3 Department of Natural Sciences, Novosibirsk State University, 630090 Novosibirsk, Russia; 4 International Tomography Center SB RAS, 630090 Novosibirsk, Russia; 5 Institute of Molecular and Cellular Biology SB RAS, 630090 Novosibirsk, Russia; 6 Novosibirsk State Agrarian University, 630039 Novosibirsk, Russia

**Keywords:** phospholipids, diet, mitochondria, metabolism, intestinal barrier

## Abstract

Dietary composition substantially determines human health and affects complex diseases, including obesity, inflammation and cancer. Thus, food supplements have been widely used to accommodate dietary composition to the needs of individuals. Among the promising supplements are dietary phospholipids (PLs) that are commonly found as natural food ingredients and as emulsifier additives. The aim of the present study was to evaluate the effect of major PLs found as food supplements on the morphology of intestinal epithelial cells upon short-term and long-term high-dose feeding in mice. In the present report, the effect of short-term and long-term high dietary PL content was studied in terms of intestinal health and leaky gut syndrome in male mice. We used transmission electron microscopy to evaluate endothelial morphology at the ultrastructural level. We found mitochondrial damage and lipid droplet accumulation in the intracristal space, which rendered mitochondria more sensitive to respiratory uncoupling as shown by a mitochondrial respiration assessment in the intestinal crypts. However, this mitochondrial damage was insufficient to induce intestinal permeability. We propose that high-dose PL treatment impairs mitochondrial morphology and acts through extensive membrane utilization via the mitochondria. The data suggest that PL supplementation should be used with precaution in individuals with mitochondrial disorders.

## 1. Introduction

Dietary composition emerges as a key factor in regulating human health that can predispose to (or prevent) complex diseases, such as metabolic syndrome, inflammation, cancer and others [1]. The most recent studies demonstrate that the Mediterranean diet is among the healthiest eating styles, whereas the Western diet is associated with multiple health risks [2,3,4]. High sugar and high fat content were found to be the most prominent risk factors both in humans and in animal models [1,5]. On the contrary, other classes of dietary compounds attenuated disease progression and improved metabolism [6,7,8]. Even fatty foods differ in their effect on human health depending on the fatty acid content of the diet [9,10]. Among the promising fatty molecules are dietary phospholipids that commonly originate from soybeans, egg yolk, milk and marine organisms [11,12,13]. PLs comprise a class of complex lipids containing a phosphate group and two fatty acid derivatives linked by a glycerol molecule [14,15]. They usually self-organize into lipid bilayers and build up cellular organelles and plasma membrane [14,15,16]. Moreover, PL molecules and their derivatives are involved in signaling pathways as second messengers and control a number of key cellular events from proliferation to olfactory signal transduction [17,18,19,20]. Multiple studies mostly agree on the overall positive effects of dietary PL supplementation on human physiology and disease progression, including cardiovascular diseases, cancer, inflammation, neurological disorders and others [21].

The major naturally occurring PL in foods is phosphatidylcholine (PC) [22]. PC consists of glycerol linked to fatty acid residues and choline as a head group and represents from 15 up to 80 percent of total PL content in PL-rich products [23]. The second most abundant PL is phosphatidylethanolamine (PE), which contains ethanolamine as a nitrogenous head group and composes about 5 to 30% of total PL content in foods [23,24]. Less abundant in dietary products are phosphatidylserine (PS), phosphatidylinositol (PI) and sphingomyelin (SM) [23,24]. PL head groups define physical and chemical characteristics of each PL. Membrane charge, curvature and shape depend on the PL content and ratio [15]. Another important trait of PLs is their fatty acid (FA) content: unsaturated FAs are mostly found in plant and marine products, whereas saturated FAs are predominant in eggs, milk and meat [11,25]. The ratio between saturated and unsaturated FAs in PLs affects membrane density and fluidity, which might be crucial to phase separation and protein docking [26,27,28]. At the same time, some PL fatty derivatives serve as signaling molecules and hormone precursors [29,30]. For instance, arachidonic acid that primarily originates from animal sources of PLs exerts proinflammatory activity, whereas eicosapentaenoic acid is found mostly in marine products and shows anti-inflammatory properties [31,32,33]. Additionally, eicosapentaenoic and another marine food-derived FA—docosahexaenoic acid—are the major precursors for endocannabinoid biosynthesis [34].

As a result of the demonstrated positive effect of PLs on human health and their multiple physiological roles, a plethora of PL-containing food supplements are available on the market [21,35,36]. PL-based supplements are available over the counter and are believed to be safe and beneficial for human health [37,38,39].The vast majority of these food supplements contain PC as the main ingredient (from 30 up to 100%), with natural soy lecithin being one of the most widespread supplements. Some vendors enrich their formula with eicosapentaenoic and docosahexaenoic fatty acids of marine origin. Additionally, soy lecithin is widely used as a dietary emulsifier in regular food processing [40,41]. Thus, modern diets contain high PL amounts of both synthetic and natural origin. At the same time, PLs are bioactive compounds and might affect the structure of membranous organelles affecting the lipid layer’s physical and chemical properties. PLs can also potentially interfere with key signaling events when ingested in high doses. However, the direct effect of PLs on cell morphology has not been fully assessed.

The aim of the present study was to evaluate the effect of major PLs found as food supplements on the morphology of intestinal epithelial cells upon short-term and long-term feeding in mice. First, we investigated the impact of PL feeding on systemic metabolism using nuclear magnetic resonance (NMR)-based metabolomic profiling of blood serum. Next, we applied transmission electron microscopy (TEM) to screen epithelial ultrastructure and, particularly, the morphology of membranous organelles such as mitochondria and membrane-containing functional complexes, such as tight and adherens junctions. We further tested whether the effect of any structural changes found in TEM had a physiologically relevant outcome regarding the function of mitochondria and junctional complexes. Our data demonstrate that both short-term and long-term supplementation of PLs in high doses affect mitochondrial morphology and function in intestinal epithelial cells.

## 2. Results

Among major PLs, PC is the most widely used in food supplements because it naturally occurs in a high proportion in dietary products and mammalian plasma membranes [22,23]. For this reason, we used PC as the major constituent in the PL feeding protocol—80% of the total dose of supplemented PLs. We also used phosphatidic acid (PA) as the precursor of all PLs at a rate of 10%. The third PL used in this study was PS, which is also common in commercial food supplements [37,42]. The PL mixture added to the animals’ food contained 10% of PS. Thus, experimental groups of mice received 34.6 g/kg in the regular mouse chow in the following composition: PC:PA:PS = 8.4:1:1.

### 2.1. Long-Term Dietary PL Supplementation Does Not Significantly Affects Systemic Metabolism

**Figure 1:**
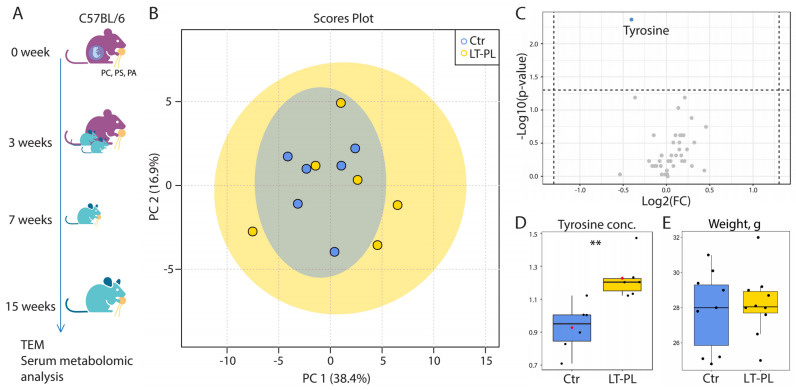
LT-PL feeding does not alter metabolic profiles of serum and weight in mice. (**A**) The scheme of LT-PL feeding. Pregnant mice were fed with PL-enriched food, their offspring had the same diet for 12 weeks. (**B**) PCA analysis of blood NMR metabolomic profiles. (**C**) Volcano plot of blood metabolites as revealed by NMR. Horizontal line depicts a cut-off at *p* = 0.05. Metabolite with differences at *p* < 0.05 (tyrosine) is shown in blue. (**D**) Median-normalized concentration of tyrosine in serum of the control and LT-PL mice (N = 6 for both groups, ** *p* < 0.01, MetaboAnalysts’s non-parametric test [43]). (**E**) Weight of PL-fed mice did not change significantly (N(Ctr) = 9, *p* > 0.05, Student’s *t*-test). Figure 1A was created with BioRender.com.

In order to evaluate the effect of the high PL diet in a long-term experiment (LT-PL group), we started supplementing pregnant dams with the PL mixture. This feeding continued until weaning, and the progeny were supplemented with PLs until the end of the experiment (Figure 1A). NMR metabolomic profiling of blood sera from PL-fed mice resulted in no significant differences between the LT-PL and the control groups as revealed by principal component analysis (Figure 1B). The Mann–Whitney U test shown in a volcano plot demonstrated no significant differences between individual metabolites, except for tyrosine (Figure 1C,D), which was significantly elevated in the LT-PL group. Moreover, the LT-PL animals’ weight did not differ significantly from that of the control animals by the end of the experiment (Figure 1E). Thus, we concluded that despite high doses of PLs, there were no major metabolic shifts upon long-term PL treatment.

### 2.2. Long-Term Dietary PL Supplementation Results in Mitochondrial Damage and Lipid Droplets Accumulation in Mitochondria

**Figure 2:**
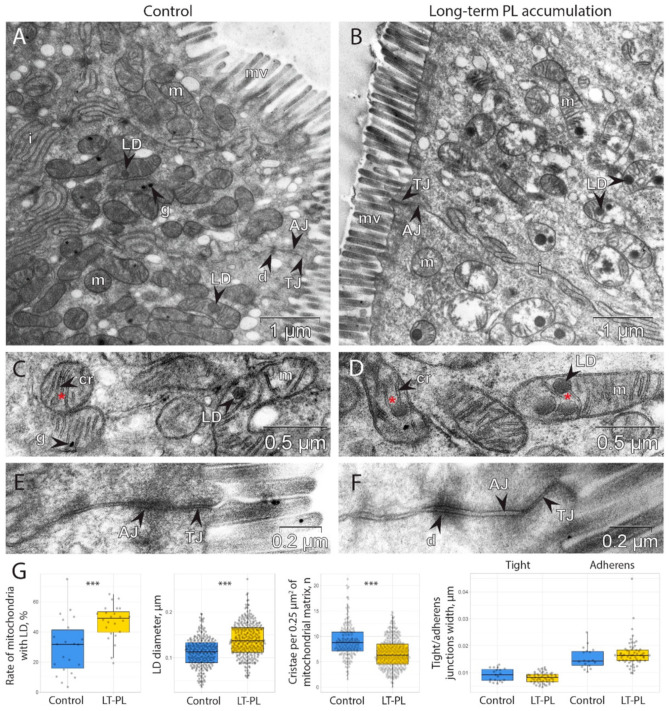
LT-PL feeding results in accumulation of lipid droplets in mitochondria and impairment of cristae in the descending colon’s epithelium. (**A**–**F**) Transmission electron microscopy of the descending colon of LT-PL mice. (**A**,**C**,**E**) Control. (**B**,**D**,**F**) LT-PL mice. Enlarged cristae are marked with red stars. AJ: adherens junction; cr: cristae; d: desmosome; g: granule; i: interdigitations; LD: lipid droplet; m: mitochondrion; mv: microvilli; TJ: tight junction. (**G**) Morphometric analysis of mitochondria, LD, AJ and TJ complexes (number of animals used for TEM analysis is 3 for both groups, *** *p* < 0.001, Student’s *t*-test).

We then used TEM to study the morphology of the intestinal epithelial cells at the ultrastructural level. We only found visible differences in the structure of the mitochondria and a significant accumulation of lipid droplets (LDs) in the intracristal space of the mitochondria and in the cytoplasm (Figure 2A,B). Closer investigation revealed that some mitochondrial cristae contained LDs within the mitochondrial matrix (Figure 2C,D). The number of cristae per square of mitochondria significantly decreased upon LT-PL treatment (Figure 2G), and many mitochondria became abnormal. At the same time, there were significantly more mitochondria with LDs in the epithelial cells of the LT-PL group and LDs were generally larger in size as compared to the control group (Figure 2G). These data demonstrate that an excess of PLs can induce LD formation in mitochondria and may impair mitochondrial respiratory function.

Next, we evaluated the structure of the tight junction (TJ) and adherens junction (AJ) complexes because mitochondrial damage is associated with the increase in intestinal barrier permeability (Figure 2E,F) [44,45,46]. However, we found no significant changes in TJ and AJ complexes upon LT-PL treatment (Figure 2G).

### 2.3. Two-Week Dietary PL Supplementation Is Sufficient to Induce Mitochondrial Damage and Lipid Drop Accumulation

**Figure 3:**
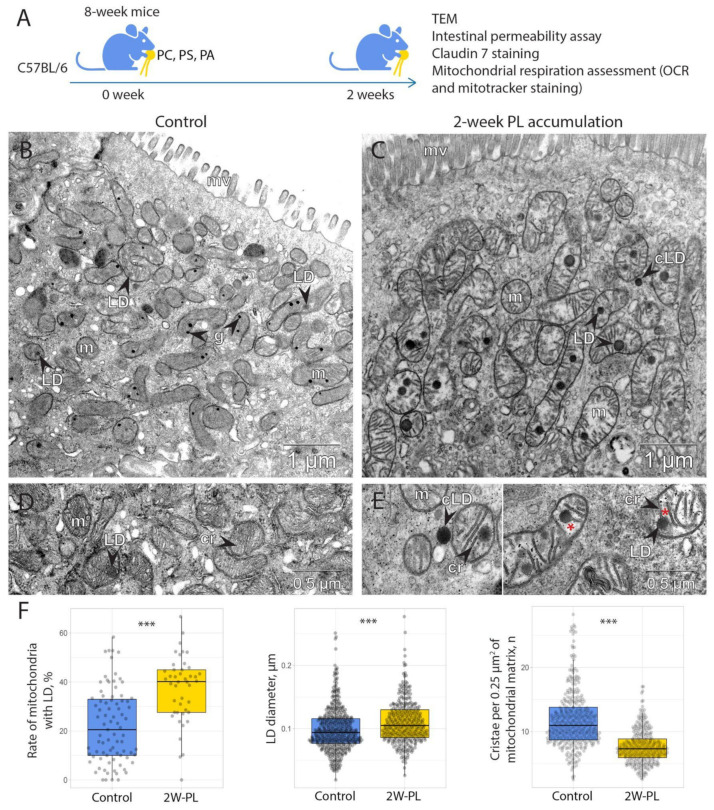
2W-PL treatment results in accumulation of lipid droplets in mitochondria and impairment of cristae in the descending colon’s epithelium. (**A**) The scheme of the 2W-PL treatment. (**B**–**E**) Transmission electron microscopy of the descending colon of 2W-PL mice. (**B**,**D**) Control. (**C**,**E**) 2W-PL mice. Enlarged cristae are marked with red stars. Cr: cristae; g: granule; LD: lipid droplet; cLD: cytoplasmic lipid droplet; m: mitochondrion; mv: microvilli. (**F**) Morphometric analysis of mitochondria and LD (number of animals used for TEM analysis is 2 for both groups, *** *p* < 0.001, Student’s *t*-test).

As long-term PL supplementation resulted in significant mitochondrial damage, we questioned whether chronic PL treatment is needed to initiate it, or if short-term feeding is sufficient to impair mitochondria. We then performed another experiment where adult mice were supplemented with the same PL-enriched diet as the ones receiving the LT-PL treatment, but for a period of two weeks (2W-PL group, Figure 3A). Intestinal samples from this experiment were analyzed using TEM in order to evaluate the structure of mitochondria. To our surprise, even two-week supplementation of PLs resulted in a strong mitochondrial damage and ultrastructural changes in mitochondrial architecture (Figure 3B–E). Morphologically, mitochondria from the 2W-PL group contained significantly fewer cristae seen within them (Figure 3F). At the same time, there were more mitochondria with LD, and the droplets were generally larger in size than the ones found in the epithelia of untreated animals (Figure 3F).

**Figure 4:**
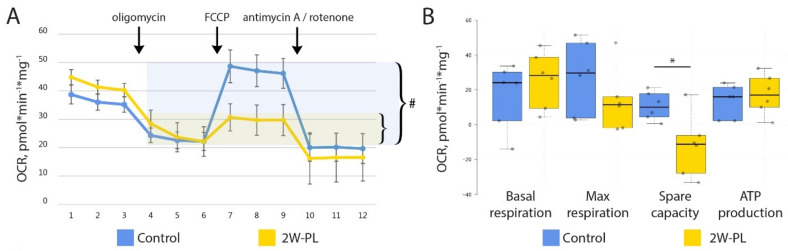
2W-PL treatment affects mitochondrial respiration. (**A**) Mitochondrial respiration measured using the Seahorse XF24 Analyzer. There was a significant upregulation of respiration after FCCP treatment in the control group only (compared to oligomycin, # *p* < 0.05, Friedman test). (**B**) Mitochondrial functional parameters as assessed with Seahorse XF24 Analyzer (N = 6 for both groups, * *p* < 0.05, Mann–Whitney U test).

Given that mitochondrial morphology was destroyed by LD formation, we questioned whether it affected their functional properties. In order to evaluate mitochondrial function, we measured mitochondrial respiration using Agilent Seahorse technology (Figure 4A). For this, we isolated intestinal crypts from the 2W-PL group and the control animals and used them directly to measure mitochondrial respiration. Our results suggest that 2W-PL mitochondria do not differ in the basal respiration; however, their respiratory capacity was negative compared to the control group (Figure 4B). As mitochondrial respiration was not fully restored in the 2W-PL crypts upon FCCP treatment, we performed an additional two-week PL feeding experiment to test cell viability in oligomycin/FCCP-treated crypts (n = 5). However, we found no significant effect of inhibitor treatment on cell viability when applied to live crypts in the same scheme and concentrations as in the mitochondrial respiration measurement (Figure S1). Taken together, these data demonstrate that an excess of PLs render mitochondria more sensitive to respiratory uncoupling by FCCP.

### 2.4. Mitochondrial Damage via PL Supplementation Is Insufficient to Induce an Increase in Intestinal Permeability

**Figure 5:**
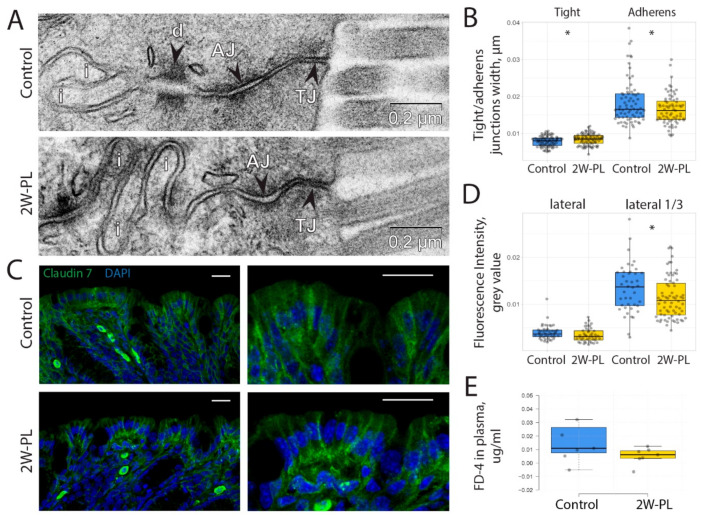
TJ and AJ morphology, Claudin-7 localization and intestinal permeability upon 2W-PL treatment. (**A**) TEM images of cell–cell apical contacts. AJ: adherens junction; d: desmosome; i: interdigitations; TJ: tight junction. (**B**) Morphometric analysis of TJ’s and AJ’s widths (N = 3 for both groups, * *p* < 0.05, Student’s *t*-test) (**C**) Claudin-7 immunostaining in the descending colon of the control and 2W-PL mice. Bar—15 μm. (**D**) Fluorescence intensity quantification of Claudin-7 along the lateral cell membrane and its apical third part (N = 3 for both groups, * *p* < 0.05, Student’s *t*-test). (**E**) Intestinal permeability assessment (N (FITC-Dextran 4kDa) = 7 for both groups, *p* > 0.05, Student’s *t*-test).

As previous findings suggest that mitochondrial dysfunction can affect intestinal barrier permeability, we tested if mitochondrial damage upon short-term PL supplementation affected TJ and AJ. Ultrastructural analysis revealed no visible striking differences in TJ and AJ morphology (Figure 5A). However, a TEM-based quantification of TJ and AJ width revealed significant differences between the control and 2W-PL groups (Figure 5B). We proposed that such differences, even though statistically significant, are unlikely to have any physiological relevance. To test this hypothesis, we used an anti-Claudin-7 antibody as a TJ marker in the intestinal epithelium (Figure 5C) and evaluated its distribution in both animal groups. This experiment revealed that there is a significant difference in Claudin-7 membrane localization between the two experimental groups: Claudin-7 apical binding in the 2W-PL group is lower than that in the control group (Figure 5D). Thus, short-term PL supplementation affects Claudin-7 binding and TJ width. However, the direct epithelial permeability assay using oral gavage of 4kDa FITC-Dextran revealed that these differences are not sufficient to impair the intestinal barrier at the functional level (Figure 5E).

Our data demonstrate that both long- and short-term PL supplementation are not completely neutral regarding the physiology of the intestinal epithelium. It induces moderate mitochondrial damage, potentially as a result of LD accumulation. Moreover, a PL overdose might affect TJ protein localization and apical junction width, which has no effect on epithelial permeability, at least in healthy animals.

## 3. Discussion

We studied the long- and short-term effects of PLs commonly found in food supplements: PC, PS and PA. All these PLs are believed to be advantageous to human health and safe to be sold over the counter. Human and animal studies support this point of view. For instance, PC supplementation demonstrated beneficial effects on intestinal inflammation in patients and in laboratory models [47,48,49,50]. Likewise, PS is regarded as beneficial for central nervous system health as was demonstrated in animal and human studies, and PS-containing supplements are widely available for everyday use [39,51,52,53]. Finally, PA can serve as a precursor in PLs biosynthesis, and it is commonly used by sportsmen to facilitate strength and muscle endurance as a mammalian target of rapamycin (mTOR) signaling pathway agonist [54,55,56].

PC and PS are of cylindrical shape and bear intrinsic propensity to form flat membranes, whereas PA’s small head group promotes it to organize into a negatively curved membrane [15]. At the same time, PS and PA are negatively charged PLs, and PC is zwitterionic in physiological conditions [57]. However, these PLs can undergo remodeling and biochemical transformations as they enter epithelial cells [58] so that they can be modified into fatty acids via head group separation or conversion into other PLs [58,59]. Therefore, the resulting PL content in the intestine may not retain the initial physical properties of these supplemented PLs.

We assume that excessive PLs and fatty acids were arranged into LDs and retained in the cytoplasm and mitochondria (Figure 2 and Figure 3). In our experiments, it remained unclear whether LDs originate from mitochondria or derive from endoplasmic reticulum (ER), as the commonly known mechanism postulates that LDs form through protein-dependent budding from ER membranes [60]. Multiple studies also suggest an active association between mitochondria and LDs to facilitate fatty acid transfer or storage in the effective vicinity to the point of lipid oxidation [61,62]. However, in these reports, LDs remain outside the mitochondrial membrane, whereas in our case, they clearly reside within the mitochondria, most often in the intercristal space. At the same time, it has been shown that LD formation in mitochondria indicates the capture and degradation of excessive membranes in a mutant mouse model [63]. The authors also demonstrate the process of LD origination within mitochondrial space in normal epithelium and conclude that it is a part of a normal detoxification process. These data clearly resemble our observations, given that externally supplemented PLs give substrates for excess membrane material.

At the same time, our data demonstrate that a high dose of PLs and, probably, excess membrane formation might impose toxicity on intestinal mitochondria both in short and chronic treatment (Figure 4). While basal respiration was the same in both groups, PL treatment rendered the mitochondria more sensitive to respiratory uncoupling. This might be due to the extensive LD formation or the general dysregulation of mitochondrial membranes upon interference with externally supplied PLs, followed by further FCCP-induced damage. The mitochondrial inner membrane is tightly regulated with regard to PL content and is specifically enriched with cardiolipin [14]. It is possible that PC, PS and PA, which are less common in the inner mitochondrial membrane, compete with cardiolipin and integrate into cristae, rendering them less effective at holding membrane potential. Cardiolipin is directly responsible for holding protons in the membrane’s vicinity due to its double-phosphate negatively charged head groups, and its replacement by other PLs may lead to the diffusion of protons and membrane depolarization [64,65].

The uptake of PLs in humans originates from diet, food additives and, for some individuals, bioactive supplements. The upper estimated dose is about 576 mg/kg daily from food additives, the average dose from the general diet is about 3.3 g per day (as an estimate of a balanced diet of 2500 kcal/day), and might increase in the case of an unbalanced Western diet [66,67]. PL food supplements may reach of about 7.5 g/day as in the case of PA intake by bodybuilders and sportsmen as per vendors’ recommendations, even though the tested safe dose is about 750 mg per day [68]. PS supplements are mostly used to attenuate age-related memory and cognitive impairments, and the suggested safe daily dose of PS is about 600 mg [37]. PC is actively tested to treat multiple disorders, such as non-alcoholic fatty liver disease at a dose of 1.8 g per day, ulcerative colitis at up to 3.2 or 4 g daily and 688 mg a day is recommended to treat brain dysfunction [50,69,70,71]. In this study, we applied the highest dose of PLs, tested safely in preclinical studies of rats [66], which is several times higher than recommended for daily intake by humans. both with a balanced diet and the recommended PL supplementation doses. Thus, the results obtained in the present study are only relevant to an extreme overdose of PLs or an imbalanced fat-enriched diet. However, our metabolomic profiling demonstrates that even these high doses of PLs are effectively absorbed in mice and do not affect systemic metabolism (Figure 1). It is most likely that the adverse effects of PLs are mostly concentrated in the intestine.

The expected effect we have also tested here is the relation between significant mitochondrial damage and intestinal barrier permeability. A number of reports, including our previous study, suggest that mitochondrial dysfunction affects TJ and AJ proteins’ localization to the plasma membrane and functional impairment of the corresponding protein complexes [45,72,73]. Here, we see some tendency for a TJ protein Claudin-7 to delocalize from the plasma membrane upon short-term PL treatment, and TJs tend to be wider in these animals. However, this tendency did not affect the functionality of TJs, as revealed by the functional permeability experiment in vivo (Figure 5). This might be due to the fact that respiratory dysfunction was moderate and did not affect baseline respiration, as was the case in our previous report involving another mouse model of mitochondrial damage or other cited studies using respiratory chain uncouplers [45,72]. Overall, this report demonstrates that high-dose PL treatment affects mitochondrial morphology and function, probably through extensive membrane utilization. This might impose new restrictions in PL intake for individuals with mitochondrial disorders. In addition, we show that mitochondrial damage does not affect intestinal permeability in mice.

## 4. Materials and Methods

### 4.1. Animals

The experiments were performed in the Scientific Research Institute of Neurosciences and Medicine (SRINM). All procedures were conducted under Russian legislation according to the standards of Good Laboratory Practice (directive # 267 from 19 June 2003 of the Ministry of Health of the Russian Federation), institutional Ethical committee guidelines and the European Convention for the protection of vertebrate animals. All procedures were approved by the Ethical committee at SRINM, protocol #3 dated 19 May 2022. All animals had SPF status, which was tested quarterly according to Federation of European laboratory animal science association’s (FELASA) recommendations [74]. The study was conducted using C57BL/6JNskrc (our in-house C57BL/6J sub-colony) strain.

Adult pregnant female mice were housed separately in individually ventilated cages (Optimice, Animal Care Systems) with birch sawdust as litter and paper cups as shelter. The housing conditions were as follows: 12 h/12 h light/dark photoperiod; and food (BioPro, Novosibirsk, Russia) and water were provided ad libitum. The 5-week-old offspring were weaned and placed in open cages in same-gender groups and housed under the same conditions.

Long-term PL treatment was performed by feeding pregnant mice with PL-enriched food starting the first day of pregnancy. The PL-enriched diet was further supplemented after weaning and continued until the end of the experiment at the age of about 12 weeks. Only male mice were used in the LT-PL experiment. PL-enriched food comprised of standard food (BioPro, Russia) mixed with PL from dietary supplement capsules in 840 mg PC (Solgar, Leonia, NJ, USA), 100 mg PA and 100 mg PS (4+NUTRITION, Padova, Italy) proportions for each 30 g of PL-enriched food. Two-week PL accumulation was performed by feeding 8-week-old male mice with PL-enriched food for 2 weeks.

Animals were euthanized by cervical dislocation. Descending colon samples were taken for TEM, OCR measurement, mitochondria staining and permeability assessments. Blood samples were taken for metabolic analyses. As for immunohistochemistry and TEM, animals were anesthetized intraperitoneally with Domitor (Orion Pharma, Espoo, Finland) at 0.25 mg/L kg body weight and Zoletil (Virbac, Carros, France) at 15 mg/L kg body weight, and perfused with 4% PFA and 12.5% glutaraldehyde solution, respectively.

### 4.2. Metabolite Extraction and Nuclear Magnetic Resonance (NMR) Spectroscopy

The metabolomic composition of the serum sample was analyzed at the Center of Collective Use “Mass spectrometric investigations” SB RAS (Novosibirsk, Russia) by high-resolution ^1^H NMR spectroscopy. The extraction of metabolites from serum was performed by using a short sample preparation protocol earlier evaluated for quantitative NMR-based metabolomics [75,76,77]. Namely, 100 μL of ice-cold methanol and 100 μL of ice-cold chloroform were added to 100 μL of serum and vortexed for 30 s, kept on ice for 10 min and incubated at −20 °C for 30 min. The mixtures were centrifuged at 12,000 rpm and at 4 °C for 30 min to pellet proteins. The top hydrophilic fraction was collected to fresh vials and lyophilized using a vacuum concentrator.

Dried extracts were re-dissolved in 600 μL of D2O containing 6 × 10^−6^ M DSS as an internal standard and 20 mM deuterated phosphate buffer to maintain pH 7.4. The 1H NMR measurements were carried out with the use of the AVANCE III HD 700 MHz NMR spectrometer (Bruker BioSpin, Ettlingen, Germany), equipped with a 16.44 T Ascend cryomagnet. The proton NMR spectra for each sample were obtained with 64 accumulations. The temperature of the sample during the data acquisition was kept at 25 °C, the detection pulse was 90 degrees. The repetition time between scans was 25 s to allow for the relaxation of all spins. Low power radiation at the water resonance frequency was applied prior to acquisition to pre-saturate the water signal. The pulse sequence zgpr was applied.

The collected NMR spectra were manually phased and baseline-corrected. Signal processing and integration were performed using MestReNova V.12 software. We used DSS at a concentration of 6 μM as reference for chemical shift and the determination of the metabolite concentration. The metabolite resonance assignments were made by comparison with the data of the Human Metabolome Database [78] (http://www.hmdb.ca, accessed on 12 November 2022) and our previous experience in the metabolomic profiling, or by adding reference compounds whenever needed. The concentration of metabolites in the samples was calculated by the integration of the peak area of a metabolite, respectively, to the DSS added to the sample. Amino acids, organic acids, alcohols, osmolytes and energy metabolism products were identified. The detailed description of metabolite quantification and validation are presented in our previous work [79]. PCA and volcano plots were performed with median-normalized metabolite concentrations using the MetaboAnalyst 5.0 web-platform with non-parametric statistics (www.metaboanalyst.ca, accessed on 12 November 2022) [43].

### 4.3. Immunohistochemistry

Mice were anesthetized intraperitoneally as described above, N = 3 per each group. Intracardial perfusion was performed using 15 mL of PBS and 15 mL of 4% PFA per each animal. Descending colon samples were post-fixed overnight in 4% PFA, then kept in 15% sucrose for 24 h and in 30% sucrose for another 24 h. Sections of 40 μm were prepared using a 550 HM Microm cryostat (ThermoFisher Scientific, Waltham, MA, USA). Sections were incubated in 1% bovine serum albumin (BSA) + 0.15% Triton X-100 in PBS for 2 h. Sections were washed three times for 5 min in PBS + 0.1% Triton X-100 (PBST). Primary antibodies to Claudin-7 (# 37-4800, Invitrogen, Waltham, MA, USA) were incubated overnight at 4 °C in PBST + 0.1% BSA and washed three times for 5 min each with PBST + 0,1% BSA. Secondary antibodies (# A-11001, Invitrogen) were incubated for 2 h at room temperature in PBST + 0,1% BSA and washed three times for 5 min each with PBST. The colonic sections were mounted in PBS with 0,15 µg/mL DAPI for 1 h and washed with PBS one time. Ready specimens were stored in a 1:1 PBS+glycerol solution on glass slides at 4 °C. For immunohistochemistry, mouse monoclonal anti-Claudin-7 (# 37-4800, Invitrogen, 1:100) was used to detect Claudin-7. Highly cross-adsorbed goat anti-mouse Alexa 488 (#A-11001, Invitrogen, 1:500) was used as the secondary antibodies.

Images were obtained using the confocal microscope LSM 710 (Carl Zeiss, Jena, Germany) with oil immersion 40×/1.3 and 100×/1.4 plan-apo lenses and the ZEN 2012 software. Fluorescence intensity quantification was performed in individual confocal slices using ImageJ software. Claudin-7 staining intensity was measured along the lateral cell membranes for their full length and apical third. At least 30 independent measurements were made for at least three biological replicates per group. Confocal microscopy was performed in the core facility of the Institute of Molecular and Cellular Biology SB RAS.

### 4.4. Transmission Electron Microscopy

Mice were anesthetized intraperitoneally as described above, N = 2–3 per each group. Intracardial perfusion was performed using 15 mL of PB and 15 mL of 12.5% glutaraldehyde (Sigma-Aldrich, St. Louis, MO, USA) per each animal. Descending colon samples were placed in a 2.5% glutaraldehyde solution in a 0.1 M sodium cacodylate buffer (pH 7.4) for 1 h at room temperature, washed and post-fixed in 1% osmium tetroxide with 0.8% potassium ferrocyanide for 1 h. Fixed samples were contrasted with 1% uranyl acetate in mQ water, dehydrated and embedded in epoxy resin (Epon 812). Semi-thin cross-sections were prepared, stained with 1% methylene blue and analyzed with an Axioscope-4 microscope (Carl Zeiss). Ultrathin (70 nm) sections for TEM were obtained with a diamond knife (Diatome, Nidau, Switzerland) on a Leica EM UC7 ultramicrotome (Leica, Wetzlar, Austria) and then examined with a JEM1400 transmission electron microscope (JEOL, Tokyo, Japan). Section preparation and TEM was performed at the Center of Collective Use for Microscopic Analysis of Biological Objects (ICG SB RAS, Novosibirsk, Russia) (FWNR-2022-0015). Fixation, dehydration and embedding were performed at the Sector of Structural Cell Biology (ICG SB RAS).

Measurements were taken in epithelial cells from the apical (lumenal) part of colonic crypts. For statistical analyses, the morphological structures were measured using ImageJ software [80] in randomly chosen sections (from 30 to 100 independent measurements per group for each structure) in a blinded manner. For TJ width assessment, three independent measurements were made per one TJ. For AJ width assessment, up to 10 independent measurements were made per one AJ. Mitochondria cristae density was calculated per 0.25 µm^2^ of mitochondrial matrix. LD diameter was measured only for LDs located inside mitochondria.

### 4.5. Crypt Isolation

The culture medium used was Dulbecco’s modified Eagle’s medium (DMEM) (Vector, Novosibirsk, Russia) supplemented with 5% FBS (Hyclone, Logan, UT, USA) and 1% antibiotic-antimycotic (ThermoFisher Scientific), hereafter referred to as the complete DMEM.

Crypts were isolated from the descending colon. Intestinal samples were gently and rapidly removed from the abdominal cavity, cut longitudinally and washed in cold PBS 3 times. The intestines were minced into 1 mm fragments, transferred in type I collagenase solution (2 mg/mL; Gibco, Billings, MT, USA) and incubated at 37 °C for 30 min. Following gentle dissociation by a pipette, incubation solutions were carefully filtered through 70 µm cell strainers and centrifuged at 250× *g* for 5 min at 4 °C. The isolated crypts were then resuspended in complete DMEM (5% FBS), and the crypt number was calculated.

### 4.6. OCR Measurement

The Seahorse XF24 Extracellular Flux bioenergetic-profiles measurement was performed as described in [81]. In short, the day before analysis, the XF 24 cartridge plate (Agilent Technologies, Santa Clara, CA, USA) was hydrated by incubation at 37 °C in a non-CO_2_ incubator overnight with 200 µm/well of XF Calibrant (Agilent Technologies).

A total of 30 min before the assay, the XF 24 cartridge plate was loaded with mitochondrial inhibitor compounds, namely 50 μM oligomycin (final concentration 5 μM), 50 μM FCCP (carbonylcyanide p-trifluoromethoxyphenylhydrazone, final concentration 5 μM) and 50 μM rotenone/antimycin A (final concentration 5 μM, all Agilent Technologies) according to the protocol provided by the manufacturer.

We prepared the Seahorse-ADF medium containing 17.5 mM glucose (Sigma-Aldrich), 2 mM L-glutamine (ThermoFisher Scientific), 1 mM sodium pyruvate (ThermoFisher Scientific) and 1% penicillin-streptomycin (ThermoFisher Scientific) in the XF Assay Medium DMEM (Agilent Technologies). Seahorse-ADF medium’s pH was adjusted to 7.4 with 3M KCl and filtered with a 0.22 µm PVDF syringe filter (Jet Biofil, Guangzhou, China).

Eight-well Seahorse XFp cell-culture microplates (#101037-004, Agilent Technologies) were pre-coated with 10 μL of Matrigel (#354277, Corning, New York, NY, USA), diluted at 1:40 with DMEM, polymerized at 5% CO_2_, 37 °C and kept at 4 °C for 24 h. On the day of analysis, Matrigel-pre-coated plates were pre-warmed at 5% CO_2_, 37 °C and used to seed 50 µL of isolated colonic crypts in complete DMEM. An aliquot of each crypt sample was used for the protein assay. The plates were used for OCR measurement with a Seahorse XF Analyzer (Agilent Technologies). After the crypts attachment to Matrigel, DMEM was replaced by 180 µm a Seahorse-ADF medium and the microplates were incubated in a non-CO2 incubator for 45 min. The Seahorse XF24 Extracellular Flux Analyzer (Agilent Technologies) was used for the assay. The program was as follows: Calibrate; Equilibrate; Loop start: 3× Mix: 3 min, Measure: 3 min, Loop end; Inject: port A (oligomycin, 50 μM), Loop start: 3× Mix: 3 min, Measure: 3 min, Loop end; Inject: port B (FCCP, 50 μM), Loop start: 3× Mix: 3 min, Measure: 3 min, Loop end; Inject: port A (Rotenone/antimycin A, 50 μM), Loop start: 3× Mix: 3 min, Measure: 3 min, Loop end; End. Crypt aliquots were collected from each sample to measure total protein with the Bradford assay and used for OCR normalization [82].

To assess cell viability in 2W-PL crypts, isolated crypts were incubated in either Seahorse-ADF medium or the same medium supplemented first with 5 μM oligomycin for 15 min with a subsequent addition of 5 μM FCCP (final concentration) for another 15 min at 37 °C. Then, crypts were resuspended into single cell suspension by shaking for 15 min at 300 rpm, stained with 0.4% trypan blue and used to calculate the dead cell ratio in an automated cell counter Countess 3 (ThermoFisher Scientific).

### 4.7. Intestinal Barrier Permeability Assessment

Intestinal permeability was measured using 4-kDa FITC-Dextran (Sigma-Aldrich, Darmstadt, Germany), N = 10 for each group. A total of 100 μL FITC-Dextran (20 mg/mL in PBS) was administered by oral gavage using a steel feeding tube. After 4 h, 200 μL of blood was collected by orbital sinus puncture. For blood collection, the eyes of the test mice were treated with a drop of an ophthalmic anesthetic (0.5% proparacaine hydrochloride ophthalmic solution, Alcon Laboratories, Alcon-Couvreur N.V. S.A., Puurs, Belgium). Blood was diluted with PBS containing 0.5% heparin and centrifuged at 250× *g* for 15 min at 4 °C. A total 100 µL of the supernatant was applied to a 96-well plate, and FITC (485 nm/535 nm) fluorescence was measured using TriStar LB 941 (Berthold Technologies, Wildbad, Germany). Baseline blood plasma fluorescence was determined in mice after oral gavage with water (N = 3 for each group) and subtracted from fluorescence obtained after FITC-Dextran gavage (N = 7 for each group). FITC-Dextran concentrations were determined from standard curves generated by serial dilutions of FITC-Dextran in PBS.

### 4.8. Statistics

The data were tested for normality using the Kolmogorov–Smirnov test. All data are presented as mean ± standard error of the mean (SEM). Normally distributed data were analyzed using Student’s *t*-test for independent samples. Not normally distributed data were analyzed using the Mann–Whitney U test. The effect of FCCP on OCR was analyzed using the Friedman test. Metabolomics data were analyzed using MetaboAnalyst software with median normalization and non-parametric statistics as suggested by the software guidelines.

## Data Availability

Raw NMR spectra, description of specimens and samples and metabolite concentrations are available at the Animal Metabolite Database repository, Experiment ID 240 (https://amdb.online/amdb/experiments/240/, accessed on 11 December 2022). All obtained data are available from the corresponding author upon request.

## Supplementary Materials

The following supporting information can be downloaded at: https://www.mdpi.com/article/10.3390/ijms24021788/s1.
