# Supplementary material for: The Effect of Dietary Phospholipids on the Ultrastructure and Function of Intestinal Epithelial Cells
Source: Int J Mol Sci. 2023 Jan 16;24(2):1788. doi: 10.3390/ijms24021788 (PMC9866517; doi:10.3390/ijms24021788)
Supplement: Supplementary file 1 [file ijms-24-01788-s001.zip › ijms-2096397-supplementary.pdf]

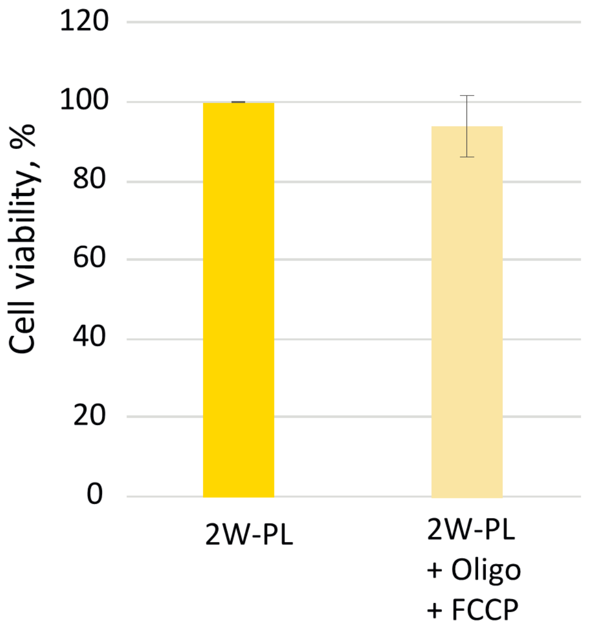

Figure S1: Cell viability assessment by trypan blue staining in live crypts upon sequential addition of oligomycin and FCCP. Untreated cell viability in each sample was taken as 100%. N = 5 in treated and untreated groups. No significant effect of treatment was found.
